# Escin’s Action on Bradykinin Pathway: Advantageous Clinical Properties for an Unknown Mechanism?

**DOI:** 10.3390/antiox13091130

**Published:** 2024-09-19

**Authors:** Gianmarco Marcianò, Cristina Vocca, Demirhan Dıraçoğlu, Rotinda Özdaş Sevgin, Luca Gallelli

**Affiliations:** 1 Operative Unit of Pharmacology and Pharmacovigilance, “Renato Dulbecco” University Hospital, 88100 Catanzaro, Italy; gianmarco.marciano3@gmail.com (G.M.); cristina_vocca@live.it (C.V.); 2 Department of Physical Medicine and Rehabilitation, Istanbul Faculty of Medicine, Istanbul University, 34093 Istanbul, Türkiye; demirhan1@yahoo.com (D.D.); rotindaozdas95@gmail.com (R.Ö.S.); 3 Department of Health Science, University of Catanzaro, 88100 Catanzaro, Italy

**Keywords:** escin, bradykinin, hippocastanum, inflammation, edema, venous insufficiency

## Abstract

Escin, extracted from horse chestnut (*Aesculus hippocastanum*) has anti-edema and anti-inflammatory effects. It is used to treat several clinical conditions, including venous insufficiency, pain, inflammation, and edema. Considering escin’s pharmacodynamic, the inhibition of the bradykinin pathway represents a particular effect, decreasing the local edema and conferring an advantage in comparison to other compounds. In this narrative review, we described the effects of escin considering its effects on bradykinin pathway.

## 1. Introduction

Escin is the active component in *Aesculus hippocastanum* (horse chestnut), a plant used for the management of several clinical conditions. The seeds of this plant contain triterpenic sapogenins. Protoescigenin and barringtogenol are the main sapogenins and their fraction has been named escin. The main isomers are ß-escin (the main constituent of the pharmaceutical preparations) and kryptoescin (less active). ß-escin is relatively water-insoluble while kryptoescin is readily water-soluble [1,2,3,4]. ß-escin displays an amphiphilic structure consisting of a hydrophobic, aglyconic part and a hydrophilic, glyconic part. The glyconic part contains glucuronic acid (GlcA) and glucose (Glc (or xylose (Xyl)) [3,5]. Despite its low water solubility, escin is administered as an oral (through selective chemical modification) and transdermal formulation for the management of inflammation, edema, thrombophlebitis, blunt lesions, hematoma, trauma, and venous insufficiency. The anti-inflammatory and anti-edematous effects of escin are related to the inhibition of the protein nuclear factor kappa-light-chain-enhancer of activated B cells (NF-κB) and hyaluronidase, as well as an increase in the glucocorticoid receptor [6].

**Figure 1:**
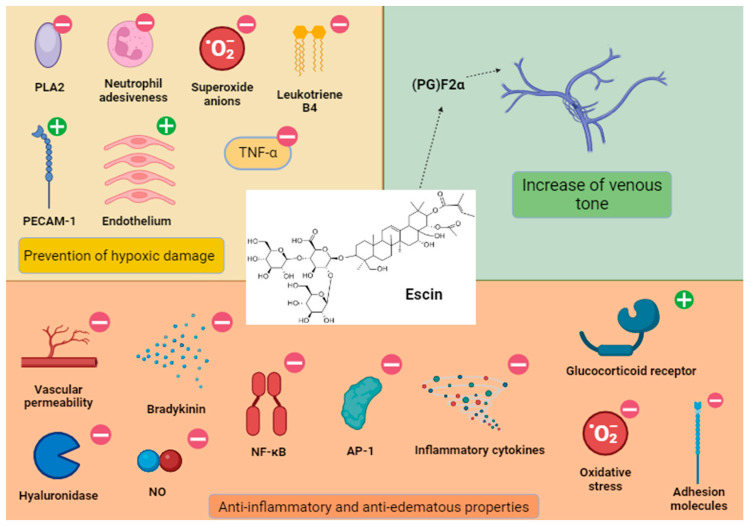
Escin antioxidant, anti-inflammatory, anti-edematous, and venotonic properties. Escin exerts its antioxidant effect, inhibiting several mediators or processes (minus sign), but promoting other molecules like PECAM-1 (plus sign) and general endothelial function. The increase in venous tone has been observed in different models and (PG)F2α is a possible mediator of this process. Inflammation is reversed by acting on the vascular level, but also on other mediators, cytokines, and transcriptional factors. Interestingly, escin displayed a glucocorticoid-like activity. AP-1, activator protein 1; NF-κB, nuclear factor kappa-light-chain-enhancer of activated B cells; NO^•^, nitric oxide; PECAM-1, platelet endothelial cell adhesion molecule-1; PG, prostaglandin; PL, phospholipase; TNF, Tumor Necrosis Factor.

In vitro studies in human umbilical vein endothelial cells (HUVECs) documented that escin protects endothelium from hypoxic damage by increasing ATP levels and reducing both phospholipase A2 expression and neutrophils adhesiveness [7]. Moreover, escin modulates the platelet endothelial cell adhesion molecule-1 (PECAM-1) expression (resulting in altered permeability) and the formation of reactive molecules (i.e., superoxide anions) and leukotriene B4 [7]. Therefore, escin reduces both permeability and proliferation of endothelium [1,8] (Figure 1). Furthermore, escin increases venous tone through a prostaglandin (PG)F2α-dependent effect. This property seems to be more effective in the early stages of chronic venous insufficiency (CVI) [9] (Figure 1).

Escin’s antioxidant effects are well described in many clinical contexts, including experimental models of non-alcoholic fatty liver disease (NAFLD), liver damage, cardiac autonomic neuropathy, and diabetic neuropathy. The activation of superoxide dismutase and the restoring of glutathione have an important role in this process [10,11,12,13,14].

Despite most of the data coming from preclinical sources, escin is widely used in clinical practice with optimal efficacy and lacking severe interactions and side effects. The most common uses are in treating phlebitis and allergic reactions, mainly after intravenous administration. The administration of escin for cutaneous application may also determine rare local reactions, whereas oral administration may rarely produce gastrointestinal side effects [1,15].

Bradykinin is a molecule involved in inflammation and swelling. It is produced through proteolytic cleavage of the kinogen (KNG) precursor (high-molecular-weight kininogen, HK) by the kinin-kallikrein system. Lysyl bradykinin (Lys-BK) is released by the action of tissue kallikrein on low molecular weight (LMW) KNGs (LKs). A plasma amino-peptidase cleaves the N-terminal Lys and liberates bradykinin as a 9-amino-acid peptide. Bradykinin seems to have major potency when compared to Lys-BK [16,17,18,19,20].

Bradykinin exerts its action through two G protein-coupled receptors named B1 and B2. The B2 receptor is constitutively expressed in different tissues (mainly in endothelium and smooth muscle), whereas the B1 receptor is expressed during inflammation. Bradykinin may determine several actions, including endothelial smooth muscle cell relaxation (vasodilation) and increases in blood flow. It seems also to be related to smooth muscle constrictions in the respiratory and gastrointestinal systems. Moreover, bradykinin, alongside PGE2, plays a role in the development of pain through the sensitization of nerve endings [21]. The B2 receptor seems to mediate the action of BK and Lys-BK, whereas the B1 receptor is activated by their metabolites (des-Arg9-BK and Lys-des-Arg9) [22].

Blunt trauma is the leading cause of morbidity and mortality in young patients and the sixth in general population. Its severity is related to the mechanism of injury and comorbidities and may result in different lesions (from contusion to fracture) [23]. Minor blunt trauma may be managed with escin to reduce pain and edema. Although bradykinin is an inflammatory mediator [18,24] and is released during coagulation [25] (with local response after trauma), no studies on its role in minor trauma have been conducted. Compounds acting on bradykinin may display an additional advantage compared to those that do not modulate this pathway. In this narrative review, we evaluate escin action on bradykinin pathways and discuss the possible benefits of this action in comparison to other substances used for similar indications.

In agreement with our recent papers [26,27,28,29], we enclosed reviews, randomized clinical trial (RCTs), or meta-analyses that evaluated the role of escin in inhibiting bradykinin pathway or described other concepts useful to this topic. We searched on PubMed, MEDLINE, Cochrane, and EMBASE databases from their inception up to 12 November 2023, using the key words “escin” or “horse chestnut” or “hippocastanum” and then “bradykinin” and “trauma” and “edema” or “inflammation” or “pain” combined with Boole’s logical operators. The records were first screened by title/abstract and then full-text articles were retrieved for eligibility evaluation. The reference lists of previous reviews and the included studies were also examined. The aim of this narrative review is to assess and describe the current evidence of escin’s action on the bradykinin pathway.

## 2. Endothelium and Inflammation

In healthy people, endothelium produces coagulation inhibitors, blocking the formation of blood clots. However, the presence of inflammation can induce thrombosis. Several inflammatory conditions have been reported to affect the endothelium, including rheumatic diseases and inflammatory bowel diseases (IBDs). In general, endothelial cell inflammation may be related to several pathways and factors, including oscillatory shear stress (unhealthy in comparison to laminar shear stress), neutrophil extracellular traps (NETs, often associated with diabetes, obesity and dyslipidemia), activation of NF-κB pathway (with the consequent increase in inflammatory cytokines and adhesion molecules) oxidative stress, and alterations of the related enzymes, toll-like receptors (TLRs) and inflammasomes [30]. Leukocytes can follow chemoattractant substances or bind to adhesion molecules to amplify the inflammation process. It is noteworthy that pro-inflammatory substances including C-reactive protein (CPR) or Tumor Necrosis Factor α (TNF-α) downregulate nitric oxide synthase (eNOS) and guanylate cyclase [31]. In a clinical study performed in 75 patients with acute myocardial infarction, Kalinskaya et al. [32] documented a correlation between CPR, interleukin (IL)-10, and impaired blood flow.

These data suggest an association between inflammation and endothelial alteration. Therefore, inhibiting this process through different pathways is a milestone in the treatment of several conditions.

## 3. Bradykinin

### 3.1. Structure and Synthesis

Bradykinin is an inflammatory mediator that exerts its action in a high number of clinical conditions including coronavirus disease 2019, asthma, neuropathy, diabetes, cancer, obesity, vasculopathy, osteoarthritis, and brain injury [33].

Bradykinin is a part of a complex pathway in the kinin-kallikrein system. It is a 9 amino-acid (Arg-Pro-Pro-Gly-Phe-Ser-Pro-Phe-Arg) peptide. There are two main pathways by which bradykinin is produced: one of them involves tissue kallikrein and LK. Tissue kallikrein is produced by different tissues, but particularly by the brain, gut, lung, pancreas, and sweat and salivary glands. Kallikrein is locally produced from prokallikrein. Then, tissue kallikrein cuts LK to produce Lys-BK (also called kallidin). This peptide is composed of 10-amino-acids (lys-arg-pro-pro-gly-phe-ser-pro-phe-arg). The N-terminal lys is then cleaved by a plasma aminopeptidase, producing BK [18,34,35].

The second pathway is more complex and is strongly related to coagulation’s cascade intrinsic pathway. Factor XII is the initiating enzyme that binds to negatively charged surfaces. It activates itself to form factor XIIa. Prekallikrein and factor XI (plasmatic substrates) are complexed with HK. FXI can bind 2 HK molecules and can release bradykinin from HK. Their binding with XIIa results in the formation of kallikrein and XIa. Then, HK (composed of 6 domains) is cleaved by kallikrein in its fourth domain at two sites to produce bradykinin. It is interesting to note that tissue kallikrein may cut both HK and LK, whereas plasma kallikrein acts selectively on HK [34,36,37].

The enzymes responsible for bradykinin’s catabolism are kininase I and II. Kininase I may act on bradykinin, producing their two metabolites des-arg9 bradykinin or des-arg10 kallidin. Kininase II is structurally identical to angiotensin converting enzyme (ACE). It is a dipeptidase that cleaves the C-terminal phe-arg from bradykinin to produce a heptapeptide, which is cleaved a second time to eliminate ser-pro, resulting in the pentapeptide arg-pro-pro-gly-phe. If the C-terminal arg of bradykinin is first removed with kininase I, then ACE acts as a tripeptidase to cut ser-pro-phe and to leave the aforementioned pentapeptide [34].

PK normally circulates as a 1:1 bimolecular complex with HK. The activation of XII factor of coagulation cascade favors the conversion of prekallikrein in kallikrein and then the formation of bradykinin alongside Hageman Factor (HF) [36].

Bradykinin has a half-life of less than 30 s and binds predominantly to kinin B2 receptors, whereas its degradation product, des-Arg9-bradykinin, binds mainly to B1 receptors [38].

### 3.2. Actions

Bradykinin exerts its action through GPCR receptors named B1 and B2. The B2 receptor is constitutively expressed in different tissues (mainly in endothelium and smooth muscle), whereas the B1 receptor is expressed during inflammation. Both the receptors determine endothelium relaxation through a classical calcium signal producing vasodilation and edema. This process involves phospholipase A2, which determines the release of PGs and eNOS.

Rex et al. [18] described the complex “modular map” of bradykinin and its involvement in inflammatory responses. The activation of bradykinin GPCRs determines the release of several mediators (e.g., protein kinase C, phospholipase C, mitogen-activated protein kinases (MAPKs) and NF-κB) and the activation of several pro-inflammatory cytokines (e.g., TNF-α, IL-1β, IL-6, IL-8).

Both bradykinin receptors are involved in injury and inflammation; in particular, the B2 receptor is involved in the acute phase of inflammation, while the B1 receptor is involved in the chronic phase. The B2 receptor has a limited role in the cellular component inflammatory response (mediated by B1) and is more frequently associated with vascular permeability, venoconstriction, and pain. In contrast, the B1 receptor promotes leukocyte migration, edema, and pain [24].

Bradykinin stimulates epithelial cells secreting compounds able to induce the chemotaxis of immune cells (macrophages and neutrophils) and the degranulation of mast cells. Pain and hyperalgesia result from the excitation and sensitization of nociceptive neurons [39].

Cambridge and Brain [40] showed that the injection of bradykinin in a rat’s knee joint induces plasma extravasation, due to the activation of B2 receptors (constitutively expressed). A possible mechanism of action of bradykinin in joints is the activation of sensory nerves, with the release of pro-inflammatory peptides and the induction of neurogenic oedema.

Bradykinin is involved in rheumatologic and orthopedic conditions (e.g., rheumatoid arthritis, gout, osteoarthritis, and psoriatic arthritis) contributing to inflammation, pain, and edema [41]. In particular, the intra-articular administration of bradykinin causes excitation and sensitization of sensory nerves, evoking pain and hyperalgesia, leukocyte recruitment, and an increase in vascular permeability and vasodilation, producing local heating and edema [41].

Seegers et al. [42] observed, in a model of rat synovitis, that B2 receptors and substance P were associated with endothelial proliferation, reverted by receptor antagonists.

Muscella and colleagues [43], in human fibroblasts derived from biopsies, observed a PGE2 increase mediated by bradykinin, through extracellular signal-regulated kinase (ERK) 1/2 and p38-dependent pathways.

It is not futile to remember that bradykinin is also responsible for angioedema onset. It is a sudden and transitory swelling of the mucous membranes and skin that may last between several hours and three days. The compromission of respiratory airways may be lethal in this context. This pathology may be hereditary (due to deficiency of C1 esterase inhibitor (C1-INH)), acquired, or related to drug consumption. It differs from histamine-induced angioedema in both clinical presentation and management [19,37].

Bradykinin is part of a complex cascade (see Introduction) in which other actors are involved. It seems to demonstrate stronger activity at the beginning of the inflammatory process, with a prevalent role in B2 receptors [44]. In consideration of the importance of bradykinin, drug discovery is focusing on B2 receptor antagonists to manage bradykinin associated disease. Currently, icatibant is the only approved drug for the management of hereditary angioedema [45,46].

There are no specific papers dedicated to the interactions between escin and the bradykinin pathway, to our knowledge. Surely, there are two important points of contact (indirect mechanism). The first is related to escin’s inhibitory action on nitric oxide (NO^•^). Conversely, bradykinin increases NO^•^, mediating vasodilation and edema [1,47]. Through this mechanism, escin may indirectly block the action of bradykinin. Furthermore, the well-documented activity of escin on the glucocorticoid receptor, resulting in anti-inflammatory activity, reduces bradykinin production, mitigating all its clinical manifestations [48].

Other possible hypotheses need to be studied further. It was observed that Horse chestnut displayed anticoagulant activity. Therefore, a theoretical inhibitory action on XIIa factor resulting in a block of prekallikrein activation to kallikrein may be considered. Nevertheless, anticoagulant activity was described mainly for the bark, and not the seeds or seed shell (from which escin is estracted) [1].

A direct action on bradykinin’s receptors seems to be improbable, considering the structural difference between the two molecules. Mechanisms of action involving HK, a direct bradykinin-escin interaction (despite the two molecules having polar bonds), or a change in receptor expression due to a direct action lack evidence. At present, an indirect mechanism of action is more probable, but new clinical and experimental studies are required to confirm this hypothesis.

**Figure 2:**
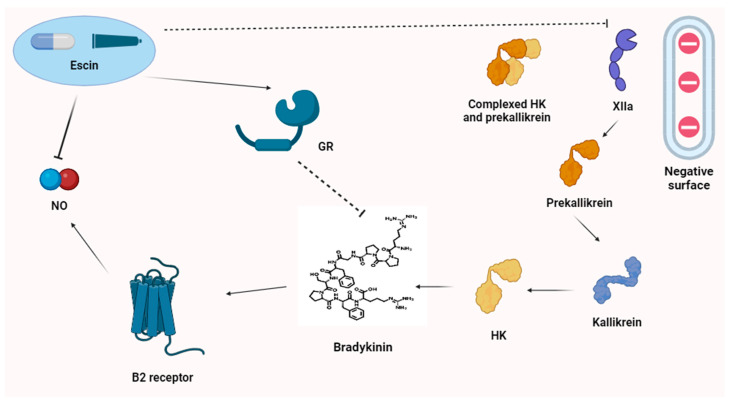
Escin’s action on the bradykinin pathway. Poor evidence exists about the precise mechanism of escin’s action on the bradykinin pathway. Three possible hypotheses may be related to (1) the action on glucocorticoid receptor, that in some models has shown to reduce the activity of bradykinin; (2) NO^•^ inhibition, an indirect mechanism, since bradykinin may raise NO^•^ concentration; (3) a possible action on coagulation cascade, since *Aesculus Ippocastanum* has shown anti-coagulant properties, but involving mainly the bark and not the seeds or seed shell. In the figure, we observe the conversion of prekallikrein in kallikrein by XIIa and then the production of bradykinin by kallikrein through its catalytic action on HK. GR, glucocorticoid receptor; HK, high-molecular-weight kininogen; NO^•^, nitric oxide; XII, activated twelve factor.

The most relevant points of contact between escin and bradykinin are summarized in Figure 2.

## 4. Anti-Inflammatory and Analgesic Effects

In an experimental model of peritonitis and pleurisy, Rothkopf and Vogel [49] showed that escin reduces the exudate and the protein permeation associated with peritonitis. In this model, escin was able to antagonize the effects of bradykinin.

Considering escin’s actions on inflammation [1,50], is legitimate to assume that it acts mainly on the B1 receptor, since the modulation of the B2 receptor cannot be ruled out. The inflammatory response prompts vasodilation, which is stimulated by NO^•^, bradykinin, histamine, and PGs. In this stage, escin counteracts the release of pro-inflammatory mediators at the vascular level, reducing inflammation [1,47]. Corticosteroids suppress the expression of bradykinin receptors in experimental models [51]. Escin may act with glucocorticoid-like activity on GR [48]. Moreover, escin may inhibit the production of PGE2 and NF-κB. Furthermore, some non-genomic effects of escin are proposed. The GR expression is increased by escin in some models, reducing inflammatory cytokines [48].

In human lung carcinoma A549 cells, Ji et al. [52] documented that β-escin sodium downregulates iNOS expression through inhibiting Janus kinase (JAK)/signal transducer and activator of transcription (STAT) signaling and p38 MAPK activation. Maghsoudi et al. [53] obtained similar results in a model of osteoarthritis in which escin was compared with ibuprofen and dexamethasone. In a clinical study performed in 156 patients with osteoarthritis, a direct correlation was documented between the expression of B2 receptors, NO^•^ levels, and the severe clinical state [54]. The increase in NO^•^ induced by bradykinin may be antagonized by escin, decreasing edema.

It is not futile to remember that escin possibly displays other anti-inflammatory mechanisms. Some of these needs better evidence to be confirmed. For example, the action on cyclooxygenases (COXs) has been described with different results. Wang and colleagues [55] observed that escin reduced the levels of matrix metalloproteinase-9 (MMP-9), TNF-α, COX-2, and PGE2 in the brain in their experimental model of poisoning-related brain edema.

Similarly, Li et al. [56] observed a statistically significant reduction in COX-2 and PGE2 in rat paw edema in experimental models managed with escin.

Conversely, other authors [57] failed to show significant escin-related activity on the COX/PGF2α pathway in rat paw edema, suggesting the more relevant role of glucocorticoid-like activity on GR. It is noteworthy that activity on GR may also result in the modulation of COX activity. The activity on GR seems to be the cornerstone of escin’s anti-inflammatory effect [48], with consequences also on the bradykinin pathway. A synergistic effect of all these mechanisms confers escin a unique advantage among natural-origin molecules.

Escin provides analgesia by acting on both B1 and B2 receptors with its anti-inflammatory and anti-edematous effects [1,47]. Few papers have been published on this topic. Nevertheless, given the effects of escin on bradykinin (inhibition), considering that inflammation induced by bradykinin is associated with pain onset [39], it is easy to understand that escin has analgesic effects also through the modulation of the bradykinin pathway.

In this setting, it is important to evaluate the role of escin in blunt trauma (associated with edema and hypoxia in the injured site), one of its relevant clinical indications. Bradykinin is released in the presence of acute trauma. Blunt trauma induces micro-vascular damage and the inflammatory mediators’ release. Despite B1 receptors being upregulated and not constitutionally expressed, B2 receptors are the main mediators of bradykinin-induced damage, through blood-flow changes, vasodilation, and the consequential formation of edema. These effects were inhibited by B2-bradykinin receptor antagonists. The inhibition of B1 receptor did not modify vasodilation and edema [38]. This suggests that the primary role of B2 receptors is in tissue damage. In this light, escin, by reducing bradykinin activity, is able to reduce the sequelae of trauma.

Bradykinin modulates nociceptors and the activation of B2 receptors determines hyperalgesia in primary sensory neurons [24], through the modulation of both receptors (e.g., α-amino-3-hydroxy-5-methyl-4-isoxazolepropionic acid (AMPA), N-methyl-D-aspartate (NMDA), transient receptor potential vanilloid (TRPV)) and channels (potassium) [58,59] (Table 1).

In an experimental study, Yi et al. [60] documented that B2 receptors are expressed in the nociceptor subpopulation, even if other actors may be involved (e.g., endogenous opioid pathway and metallo-endopeptidases) [27,61,62] (Table 1).

**Table 1:** Experimental studies on bradykinin’s role in pain.

Authors	Year	Findings
Steranka et al. [63]	1988	Bradykinin antagonists reduced vasculogenic pain and hyperalgesia in both models of acute and chronic pain. Bradykinin receptors were localized in substantia gelatinosa, dorsal root, and trigeminal ganglia of guinea pigs.
Wang and colleagues [64]	2005	Experimental model of spinal cord pain. Bradykinin, through B2 receptors, activates α-amino-3-hydroxy-5-methyl-4-isoxazolepropionic acid (AMPA) and N-methyl-D-aspartate (NMDA receptors), inducing hyperalgesia. Pain was determined by bradykinin increase and the administration of an intrathecal B2 antagonist reverted central sensitization.
DomBourian et al. [58]	2006	Experimental model of spinal cord injury: the activation of B1 receptor resulted in increased calcium influx and neural activation through transient receptor potential vanilloid (TRPV1) modulation.
Luo et al. [61]	2008	Dynorphin A (an endogenous opioid) activates B2 receptor even in absence of bradykinin increase inducing hyperalgesia.
Gomez and colleagues [62]	2011	The inhibition of EP24.15 and EP24.16 metallo-endopeptidases in increases the B2 receptor activation by bradykinin.
Choi and Hwang [59]	2018	The role of bradykinin in modulating TRPV and other ion channels TRPVA, Anoctamin (ANO) 1 (calcium activated chloride channels) with possible consequences in pain onset. Furthermore, bradykinin seems to inhibit potassium channel resulting in depolarization.
Yi and colleagues [60]	2024	Chronic pain increases B1 receptors, while acute exposure to bradykinin increased neuron excitability.

## 5. Blood Circulation and Coagulation

Dudek-Makuch and Studzinska-Sroka [65] report an effect of escin on bradykinin in the setting of venous insufficiency. Escin produces a selective sensitization of vascular smooth muscles to calcium ions and reduces capillary permeability antagonizing the action of bradykinin. In this way, it provides antiedematous and venotonic effects [1,47]. Despite the lack of clinical data concerning escin’s venotonic effect through bradykinin modulation, it is possible to assume that bradykinin could be a possible target due to its role in venous insufficiency (see the sections below).

Bradykinin has a role in the modulation of venous capacitance. Gunaruwan et al., in 50 patients (20 health volunteers, 16 with heart failure treated with ACE inhibitors, and 14 with heart failure treated with angiotensin receptor blockers, ARBs) showed that intra-arterial infusion of bradykinin increased unstressed forearm vascular volume in all patients, particularly in those treated with ACE inhibitors. Blood flow increased in all patients, but less in patients using ARBs. The infusion of receptor antagonists reduced blood flow and basal unstressed volume only in ACE inhibitors patients. Therefore, bradykinin contributes to venous tone only in chronic heart failure patients managed with this drug class. Bradykinin seems to have also a role in venous insufficiency [66]. The activation of phospholipase A2 determines the production of PGs, bradykinin, and other mediators. Pain in venous disease seems to be mediated by bradykinin, since bradykinin increases pain perception in healthy subjects by intravenous or perivenous application. The action of bradykinin appears to be related to NO^•^ release by endothelial cells. PG E2 seems also to amplify this process. A complex inflammatory cascade is activated in chronic venous disease, progressively leading to edema and modification of the veins’ structure in the late stages of this clinical condition [67].

Other effects of escin with respect to NO^•^ inhibition and GR modulation may be supposed. *Aesculus hippocastanum* may increase the hemorrhagic risk determining an increase in international normalized ratio (INR) [1]. This effect could be related to the coumarin derivatives (aesculin and fraxetin) present in the bark of *Aesculus*, which may be able to modulate the factors II, VII, IX, and X [68]. The derivatives are not present in the seeds or seed shell, the parts from which escin is extracted [1], therefore the use of escin does not induce the development of bleeding. Escin displays also a protective action on endothelium modulating PECAM-1 expression and reducing the formation of reactive molecules. It also reduced endothelium proliferation [3].

## 6. Comparison with Other Natural Products

*Boswellia dalzielii* is a plant known for decades containing several pharmacologically active substances, including flavonoids, terpenoids, saponins, and steroids, among others, with effects in rheumatoid arthritis or inflammatory conditions [69]. In an experimental study by Mbiantcha et al. [70], *Boswellia* reduced the inflammation induced by several substances including carrageenan, arachidonic acid, histamine, serotonin, PGs, and bradykinin. Despite these results, the substances (arachidonic acid, histamine, serotonin, and PG E2) were administered to the same animal. Therefore, the results may lack specificity, even if the authors describe a good efficacy of the extract in reducing paw inflammation induced by bradykinin.

Bromelain is a group of proteolytic enzymes contained in pineapple or *Ananas comosus*. The oral administration of bromelain has been shown to have anti-cancer, anti-microbial, antithrombotic and fibrinolytic, antiviral, anti-edematous, and anti-inflammatory effects [71]. Bromelain reduces bradykinin levels (by about 60%) in inflamed tissues [71,72].

Diosmin, a flavonoid extracted from citrus fruit, is often prescribed for the management of hemorrhoids, leg ulcers, venous insufficiency, varicose veins, and other circulatory diseases. It is often co-administered with hesperidin, another substance of natural origin. These compounds may reduce inflammation and restore blood flow [73]. According to Nicolaides [74], diosmin possibly inhibits bradykinin-induced leakage in the context of leg edema. However, the experimental proof of this assumption is limited to rat models [75], where the authors documented an improvement in permeability, but not in leakage.

Escin reduces the activity of bradykinin pathway, which may offer a relevant advantage in comparison to other substances which also display anti-inflammatory activity. Bromelain, diosmine, and *Boswellia* seem to exert poorly documented effects on the bradykinin pathway. However, the superior anti-inflammatory activity of escin, due to its action on GR, confers this drug a further advantage.

Despite these observations, no comparative studies of escin (a natural drug) and the cited nutraceutical exist. Overall, escin appears like a drug with an excellent safety profile and versatile pharmacodynamics. These allow it to obtain optimal anti-inflammatory and antiedema effects, also according to bradykinin pathway suppression. Nevertheless, human studies are necessary to evaluate the effect of escin on bradykinin.
